# Regulatory T Cells in Respiratory Health and Diseases

**DOI:** 10.1155/2019/1907807

**Published:** 2019-11-20

**Authors:** Rani Singh, Daniel Alape, Andrés de Lima, Juan Ascanio, Adnan Majid, Sidhu P. Gangadharan

**Affiliations:** Division of Thoracic Surgery and Interventional Pulmonology, Beth Israel Deaconess Medical Center, Harvard Medical School, Boston, MA, USA

## Abstract

Respiratory diseases compromise the health of millions of people all over the world and are strongly linked to the immune dysfunction. CD4+FOXP3+ T regulatory cells, also known as Tregs, have a central role maintaining tissue homeostasis during immune responses. Their activity and clinical impact have been widely studied in different clinical conditions including autoimmune diseases, inflammatory conditions, and cancer, amongst others. Tregs express transcription factor forkhead box P3 (FOXP3), which allows regulation of the immune response through anti-inflammatory cytokines such as IL-10 or transforming growth factor beta (TGF-*β*) and direct cell-to-cell interaction. Maintenance of immune tolerance is achieved via modulation of effector CD4+ T helper 1, 2 or 17 (Th1, Th2, Th17) cells by Tregs. This review highlights the recent progress in the understanding of Tregs in different disorders of the respiratory system.

## 1. Introduction

The role of regulatory T cells (Tregs) and their different subpopulations in pulmonary diseases, has been studied extensively. Imbalances in these subpopulations have been linked to changes in clinical outcomes in different pulmonary conditions, including chronic obstructive pulmonary disease (COPD), asthma, sarcoidosis, pulmonary fibrosis, and lung cancer [1–3]. Changes in the local inflammatory milieu may add to altered Treg concentration and/or function in the airways. While in the abundance of regulatory T cells can have a detrimental effect in allergic airway hyperreactivity [4]; adoptive transfer of iTreg cells can inhibit allergic inflammation and hyperreactivity [5, 6]. Similar patterns have also been observed in studies from mouse models [3, 7, 8]. Evidence suggests that variations in cell-mediated responses, regulated by regulatory T-cells, have a direct role on the pathogenesis of different airway diseases that arise following noxious stimuli exposure [9].

Here we review some of the mechanisms associated with physiological and pathological states of this cell type. A summary of latest findings in relation to the maintenance of protection from pulmonary disorders including severe asthma, chronic obstructive pulmonary diseases (COPD), lung cancer, idiopathic pulmonary fibrosis, lung cancer and sarcoidosis, is included.

### 1.1. Overview of Tregs in Physiological and Pathological States

Tregs constitute a small subpopulation of CD4+ T cells that maintain homeostasis under multiple immune scenarios. These cells develop either naturally (nTregs, developed in the thymus) or after peripheral induction (iTregs, arising in peripheral circulation from conventional T cells). They are characterized by the presence of CD4 and CD25 surface biomarkers, account for 1–4% of circulating CD4+ lymphocytes [10] and often exhibit a high degree of cell plasticity, regulated by a complex network of transcription factors [11]. Although several details in the role of Tregs remain to be described, their activity occurs primarily via modulation of effector T cell activity, allowing optimal function of the T-cell receptor signaling process [11].

Aberrations in function or count of Tregs are a feature of autoimmune diseases and amplifying functional Tregs ex vivo makes them ideal nominees for autologous cell therapy to treat human autoimmune diseases [12]. The expression of transcription factor forkhead box P3 (FOXP3) allows Treg cells to counterbalance autoimmune responses through secretion of a series of anti-inflammatory cytokines, including IL-10 and transforming growth factor beta (TGF-*β*) [10]. Although Treg cell populations are generally stable [13], during inflammatory or pathologic conditions some subpopulations become unstable, diminish FOXP3 expression and further differentiate into common effector T cell population subtypes (termed “ex-Treg cells”) [14]. Unstable Treg populations are characterized by a low CD25expression and may represent a newly developed cell lineage that is not entirely dedicated to the Treg lineage, but still retains certain plasticity [15, 16].

Other molecular pathways associated with decreased stability and changes in FOXP3 expression in pathological states include suppressor of cytokine signaling 1(SOCS1), which is highly expressed in Tregs and plays a role in maintaining suppressive functions of nTregs [17, 18]. Suppressive role, particularly over Th2 and Th17 responses have been proposed to involve GATA3- and ROR*γ*t- gene expression [19]. The relation between Tregs and Th17 cells is mediated by certain cytokines, including IL-6 which induces naïve T cells into the Th17 phenotype [20].

Tregs have several membrane receptors that allow chemotaxis, for instance, CCR4, CCR5, CCR7, and CCR8 have been identified as important in the recruitment to either the lung or lymphatic tissue, where regulatory action following allergen challenge is performed [21]. Other compounds, such as sphingosine 1-phosphate (S1P) immunomodulator receptor agonist (FTY720) are also involved in Treg chemotaxis. Activation of this molecular pathway results in an increased sequestration of regulatory T cells in the spleen, reducing recruitment at sites of inflammation [22]. These molecules are often thought as potential therapeutic targets in an attempt to manipulate migration of Tregs to the lungs, limiting inflammation.

**Figure 1:**
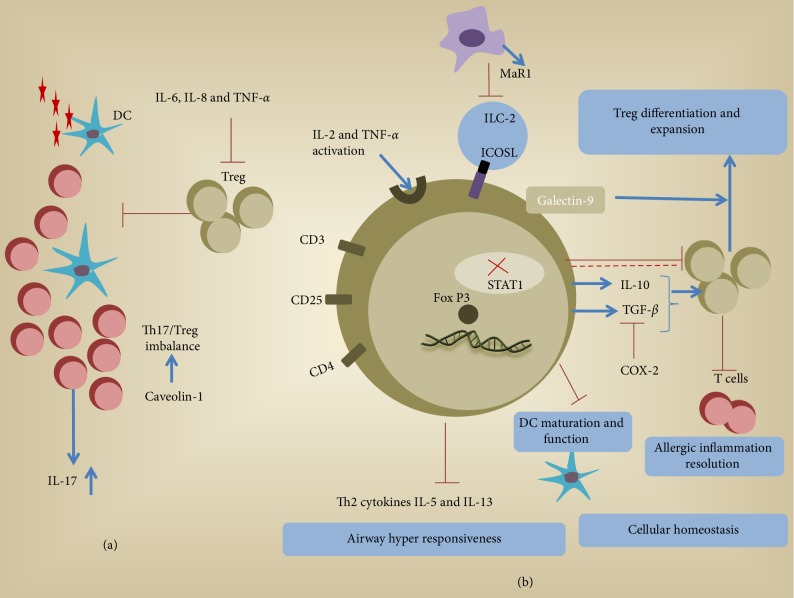
Potential mechanistic role of Treg function in airway inflammation. (a) An unknown airborne antigen activates dendritic cells. Infiltration of T cells, Th17 (1L-17 production) and Tregs cells at the site, may sometimes cause imbalance in Th17/Treg population. Cytokines like IL-1*β* and IL-6 may inhibit TGF-*β* production and can further downregulate Treg activity. (b) Higher TNF-R2 expression mainly due to IL-2 and TNF-*α* activation is associated with sarcoidosis. ICOS-L on ILC2 engages with ICOS on Tregs enhancing immune regulation. Optimal level of STAT1 within the cell is required for proper regulation of Th1 cells by Treg lymphocytes. Circulating T regs inhibits TH2 cytokine production that otherwise leads to uncontrolled proliferation of pro-inflammatory cell lineages and airway hyperresponsiveness. Increased levels of TGF-*β* and IL-10 are compatible with increased Tregs. Regulatory T cells also express galectin-9 that can limit the adaptive immune response, in particular, T cell response, while promoting the expansion of regulatory cells. Inflammatory cytokines such as TNF-*α* and IL-6 can act as a driving factor for the generation of IL-10-producing Tregs through ICOS/ICOS-L interactions. Therapeutic strategy for allergic inflammation that engages MaR1-conditioned Tregs to control ILC2 and CD4+ T cell effector functions. Alternatively, specific regulatory T cells can be suppressed with pleiotropic cytokine Activin-A and acts as a critical controller of allergic airway disease and also suppresses Th responses through regulation of DC function and decreased DC maturation.

In summary, Tregs have different effector mechanisms that act conjunctively to attain balance between different T cell subsets (Th1, 2, and 17) (Figure 1). Dysfunction at any level of the different biomolecular pathways within the regulatory cell population may lead to an overactive immune system. A summary of prominent Treg studies in both mice and human is provided in Table 1. Such imbalance eventually causes further development of effector responses to harmless antigens leading to the development of inflammatory conditions in the lungs.

## 2. Regulatory T-Cells in Pneumonia

About over one million people seek medical attention for pneumonia and other complicated lower respiratory tract infections yearly in the United States [23]. Despite advances in health care policies, vaccinations and antibiotic therapies, amongst others, it is still a major cause of morbidity and mortality [23]. Pneumonia is most commonly by viral or bacterial agents, although fungal and parasitic species may also be responsible. Recent efforts have been made in trying to understand immunomodulatory mechanisms behind the pathogenesis of pneumonia.

T cells play a critical role in pulmonary host defense against pathogens. Inadequate T cell response, as demonstrated by imbalances in T cell subpopulations, can lead to increment in the susceptibility and infection dissemination, as demonstrated in animal models. Some of the proposed mechanisms behind this theory include potential lung tissue injury secondary to unregulated T cell activity [24]. Treg depletion has been demonstrated to play an important role in the pathogenesis of Chlamydia pneumonia infection via antigen sensitization [25]. Also, this population plays a protective role against pneumococcal pneumonia through mechanisms that involve TGF-*β* pathways. This resistance may be enhanced by administering T regulatory cells or inhibited by blocking the activity of such [26]. Interestingly, Tregs do not have a direct impact in the host response against *Pseudomonas aeruginosa* pneumonia [27]. Other studies have used respiratory syncytial virus models to reveal that depletion of Treg-cell may result in delayed migration of CD8^+^ T-cell subpopulations [28]. Similar results have been seen in studies using Influenza A virus models in mice, were infected individuals present an intense induction of Foxp3(+) CD4(+) T cells. However, no significant impact in mortality, viral clearance or lung tissue cellularity has been demonstrated [28, 29].

Despite promising results in animal and in vitro models, the lack of clinical data in human studies limits therapeutic applications. In the future, more studies are expected to help determine how these new findings can be used as a therapy to decrease the pathogen burden.

## 3. Role of Tregs in Pulmonary Parasitic Infections

Parasitic infections of the lung occur in both immunocompetent and immunocompromised patients and may affect the respiratory system [30]. It is known that parasitic infections can cause gastrointestinal, respiratory, and ophthalmologic disease. Human African trypanosomiasis (HAT) or sleeping sickness is caused by extracellular protozoan parasites belonging to *Trypanosoma* sp. and HAT remains a risk to more than 60 million people in 36 sub-Saharan countries. Significant thickening of the bronchial walls accompanied by inflammatory reactions has been observed due to *Trypanosoma* sp. parasite infiltration in animal infection models [31]. These pathologies can cause lung damage causing pulmonary alveolar hemorrhage, bronchiolitis, and pneumonitis and even pulmonary hypertension caused by *Trypanosoma cruzi* [31]. Our recent work has shown that low dose *Trypanosoma congolense* infection can enhance cytokines such as interleukin-10 (IL-10), IL-6, IL-12, tumor necrosis factor alpha (TNF-*α*), transforming growth factor *β* (TGF-*β*), and gamma interferon (IFN-*γ*) by spleen and draining lymph node cells that can display increased T regulatory cells which leads to increased susceptibility to reinfection to parasitic infection in animal model [32]. Also, we reported that depletion of Tregs by anti-CD25 monoclonal antibody treatment during primary infection or before challenge infection resulting in repeated low-dose infection completely eliminated the low-dose-induced enhanced susceptibility [32].

Also, parasites such as *Strongyloides stercoralis, Schistosoma haemaetobium, and Echinococcus* spp. have been associated with down-regulation of T lymphocyte functions including enhancement of T cell apoptosis and self-inactivation via a T-cell exhaustion phenomena [33]. *Strongyloidiasis* is more common in tropical countries. This nematode penetrated the skin as filarform larvae, enter to the blood stream and migrate to heart and lungs. Once in the lungs they migrate to alveoli to subsequent ascent to the airway up to the upper gastrointestinal tract to finally being swallowed and settle in the small intestine [30]. Studies in mice have shown that Foxp3^+^ Treg numbers increase rapidly during infection with the nematode *Strongyloides ratti*. Also, same study showed that the infection by *S. ratti* dramatically reduce when Tregs are suppressed [34].

*Schistosomes* species such as *S. mansoni, S. haematobium, and S. japonicum* have been also associated with pulmonary infections. Humans are infected by cercariae during contact with fresh water. The organisms enter the circulation and pass through the heart, lungs, and then the liver to reach the target venous plexus. Chronic schistosomiasis in the venous plexus can cause hepatosplenomegaly and portal hypertension that can lead increased pressure in the venous pulmonary system and cause pulmonary hypertension. Current literature has proved that elevated levels of Tregs lymphocytes have been reported during chronic human schistosomiasis. An ex-vivo study in peripheral blood monuclear cells (PBMC's) of *S. mansoni*-infected individuals concluded that the removal of Treg from the mononuclear cells leads to increased levels of phytohemagglutinin (PHA)-stimulated interferon gamma (IFNγ) production and decreased interleukin-10 (IL-10) responses. In the past, IL-10 has been correlated with control of pathogenesis, reduction of morbidity and prolonged survival in human schistosomiasis [35]. The authors also found that Foxp3+ Tregs appear to be one of the key players in immune-regulatory processes favoring metacestode survival by affecting antigen presentation and suppressing Th1-type immune responses [35].

Other common worldwide distributed parasite that has been related with pulmonary involvement is Hydatid disease caused by larvae *Echinococcus* tapeworm species with dogs being the definitive host. However, when humans become accidental intermediate host after eating food contaminated with eggs, the larvae migrate from the gastrointestinal tract to the bloodstream to finally migrate to the liver. Although most cysts form in the liver, 20–30% form in the lung causing a lethal disease [30]. A study using the spleen of mice infected by *Echinococcus* there was evidence of Foxp3 and IL-10 mRNA expression increment. The authors suggested that suggest that affecting Foxp3^+^ Tregs could offer an attractive target in the development of an immunotherapy against AE.

### 3.1. Role of Tregs in Sarcoidosis

Sarcoidosis is a systemic granulomatous disease that often affects the thoracic cavity. Nonnecrotizing granulomas are formed that are primarily characterized by type 1 T-helper cells and giant multinucleated cells [36], are stimulated by an uncontrolled T cell-mediated inflammatory response to an antigen [37]. Traditionally, sarcoidosis patients exhibit a predominance of Th1 type cytokines. Hence, adequate disease control often depends on the activity of highly potent Tregs [38]. Individuals who lack sufficient numbers of suppressor cells to modulate the function of lung T helper cells, are at high risk of developing deteriorating pulmonary function [39]. Imbalances between circulating or airway Th17 cells and Tregs cells may be involved in the pathogenesis of sarcoidosis. However, this subject remains controversial in current literature [40–42]. Increased levels of Treg cells in bronchoalveolar lavage fluid (BALF) and sputum samples from patients with active pulmonary sarcoidosis have been described [40]. Yet, these cells often exhibit poor suppressive ability and anergy, as demonstrated by lower levels of IL-2 and IFN-*γ* [43]. Others have reported decreased Tregs and increased Th17 cells in peripheral blood and BALF samples in this patient population [41]. Individuals who progress into chronic stages of the disease often exhibit reduced Treg counts in BALF, different from those who achieve spontaneous resolution [44].

Alterations in Treg counts in sarcoidosis patients are attributed to a dysregulated signaling network among Tregs and Th1 cells which include pathways associated to JAK/STAT interactions and TGF-*β*, IFN-*γ* and IL-2 cytokines [36]. Optimal levels of signal transducer and activator of transcription factor 1 (STAT1) within the Treg cell are required to regulate Th1 cells [36]. IL-10 and TGF-*β* are also induced by inducible T-cell costimulatory phenotype (ICOS1), which is highly expressed in Treg subpopulations isolated from BAL samples during periods of high disease activity. Since ICOS1^hi^ favors immunomodulation, it has been proposed as a potential target for disease control in this patient population [45].

Other molecular targets that have been identified to be potentially dysfunctional in Tregs from patients with sarcoidosis include ICAM-1 and Galectin-9. Studies in mice have demonstrated that ICAM-1 deficient Tregs are unable to produce adequate levels of IL-10, hence favoring the granuloma formation [46]. Similarly, a dysfunctional interaction between membrane proteins Galectin-9 and T-cell immunoglobulin and mucin-domain (TIM)-containing molecules, in regulatory T cells is associated with an exaggerated Th1 response [47]. Other deficiencies in Tregs related to sarcoidosis development include mutations in genes BTNL2 and ANXA11 whose gene products have anti-inflammatory and immune regulatory properties via modulation of tumor necrosis factor [48]. Interestingly, mutation in ANXA1 impairs apoptosis of inflammatory cells which leads to persistent inflammation and maintenance of granulomas [48, 49].

Several treatment strategies have been proposed for sarcoidosis. First-line therapies are based on systemic steroids, second-line agents include methotrexate (MTX) and azathioprine and third-line options consist of monoclonal antibodies targeted specifically at tumor necrosis factor-alpha (TNF-*α*) [50]. Therapy with systemic corticosteroids enhances suppressor lymphocyte activity as demonstrated by their increased adherence to macrophages [42]. Similarly, therapies that target inhibition of TNF-*α* compensate the deficiency in Treg count and differentiation [51]. Infliximab therapy diminishes Treg count and lowers TNF receptor 2 expression on Tregs, originally increased in sarcoidosis [52]. Increased understanding about their mechanisms of action may add to the quest for new therapeutic targets.

## 4. Role of Tregs in Chronic Obstructive Pulmonary Disease

Chronic obstructive pulmonary disease (COPD) is characterized by chronic inflammation and destruction of the small airways and lung parenchyma [53]. Complex immune pathological processes involving several cell populations from different lineages have been described in the development of this disease [54]. Regulatory T cells have a central role in the pathogenesis of COPD [55]. Although their presence in the airways of patients with COPD has been documented [55, 56], controversy still remains when determining subpopulation changes during pathological states. Different studies analyzing cellular populations in BAL fluid samples have described decreased counts of regulatory T cells in the airways of patients with COPD [56, 57]. However, others have reported higher levels of Tregs in BAL fluid from similar patient groups [58]. Interestingly, regulatory T cells from bronchial samples, when present in excessive amounts, have impaired regulatory function, as demonstrated by a decreased expression of FOXP3 and CD27 biomarkers [58, 59]. This phenomenon is related to reduced levels of TGF-*β* in the airways of these patients, which induces the immunosuppressive activity of Treg cells [56]. Decreased regulatory T-cell activity may also explain why BAL samples from smokers and COPD patients have higher proportions of pro inflammatory B and T cell populations [56].

The relation between different circulating regulatory T cell subpopulations is often altered in COPD patients [55]. These populations also demonstrate impaired function as they fail to suppress CD4+ T-cell activation when stimulated [60]. Abnormally high circulating immunological markers, including C-reactive protein, IL-6, IL-8, and TNF-*α* have been associated with decreased pulmonary function in COPD patients [61]. Alterations in the expression of regulatory protein Caveolin-1 play a crucial role in the imbalance between Th17 and Treg populations in this patient group [62]. Patients with smaller proportions of resting regulatory Tregs and higher counts of cytokine secreting T-cell subtypes, are associated with increased IL-17 and IFN-*γ* concentrations [55]. Decreased Treg counts and FoxP3 expression have been correlated with increased COPD severity [63]. Alterations in the relation between regulatory T cells and Th17 cells occur particularly during acute COPD exacerbations, when substantial increases in IL-17 are observed [64].

Paradoxically, others have described increased activity of Tregs in COPD patients, as demonstrated by increased levels of circulating TGF-*β* and IL-10 [65]. This suggests that regulatory T cells may have a dual role in the pathogenesis of COPD. While deficient Treg activity may lead to chronic inflammation caused by unopposed effector cells, Treg overactivity may be responsible for a permissive immune system that favors pathogen colonization of the airways [65]. These processes are also present in extrapulmonary tissues, including lymphatic nodes. Increased regulatory T-cell counts in pulmonary lymphoid follicles lead to a higher suppression of cell mediated immunity (involving CD8+ effector T cells) [66]. Such alterations account for the higher risk of recurrent exacerbations that are caused by bacterial infections in COPD patients [66].

From a pharmacological standpoint, imbalances in regulatory T cell populations in COPD patients may be modulated when different therapies are applied. In general, Treatment with inhaled *β*2-agnosits, anticholinergics and steroids decrease the level of pro-inflammatory cytokines (IL-8, IL-17 and TNF-*α*) in COPD patients [67, 68]. This effect is attributed to an increase in the population of circulating and bronchial regulatory T-cells and a decrease in the counts of other effector T cell populations [67, 68].

Functional alterations in regulatory cell population lead to immunological dysfunction, as evidenced by abnormal changes in levels of different pro-inflammatory and regulatory cytokines. These alterations are further confirmed by changes in the relation between regulatory, myeloid-derived suppressive and effector T cells [65, 69]. Consequently, COPD patients often have abnormalities in the airway microbiome [70] which may increase the risk of frequent exacerbations due to infections [65]. Altogether, regulatory T-cells likely play an important role in the pathogenesis of COPD. Controversy still exists regarding how this cell population may be increased or decreased during certain periods of the entire disease course.

## 5. Regulatory T-Cells in Asthma

Asthma is characterized by chronic airway inflammation that leads to a variety of symptoms that are often reversible and have variable intensity and duration [71]. Although different phenotypes have been described, allergic asthma is the most common. In this condition, allergen exposure triggers an exaggerated Th2 response which unchains severe airway inflammation and bronchial wall hyper-reactivity [72]. This exaggerated response is partially attributed to a defective Th2 modulation by regulatory T-cells and an ineffective interaction between Tregs and antigen presenting cells [5, 73–75].

Circulating Tregs from individuals with allergic phenotype asthma have a reduced ability to inhibit production of Th2 cytokines (IL-5 and IL-13). This defective regulation of the Th2-type immunity is responsible for an uncontrolled proliferation of proinflammatory cell lineages and airway hyper responsiveness following allergen exposure [76, 77]. Interestingly, equal or higher proportions of circulating regulatory T cells have been described in this patient population [78, 79]. Despite high counts, these cellular populations have a limited inhibitory activity, as demonstrated by reduced FOXP3 expression [78, 79] and decreased chemotactic response (when stimulated with CCL1) [80]. Suboptimal chemotactic response in this cell population is correlated with symptom severity referred by patients [80]. Additionally, regulatory T cells isolated from BAL and peripheral blood from patients with allergic asthma have lower expression of XCL1 and XCR1, which is associated with a suboptimal inhibitory function [81].

Defective regulatory T-cell activity plays a leading role in allergen-induced inflammation as these cells can modulate airway eosinophilia, Th2 cellular subpopulations and cytokines (primarily IL5, IL13, and TGF-*β*) [5]. Patients with atopic asthma have decreased regulatory cell counts in bronchoalveolar lavage fluid, likely why the regulatory activity over effector cells is suboptimal [82]. In cases of asthma associated with occupational exposure, increased bronchial density of regulatory T cells have been identified. However, these have been described along with higher concentrations of effector T cells, proliferative T cells and activated CD8+ cells, suggesting that the inhibitory role of these cells is ineffective [83].

**Figure 2:**
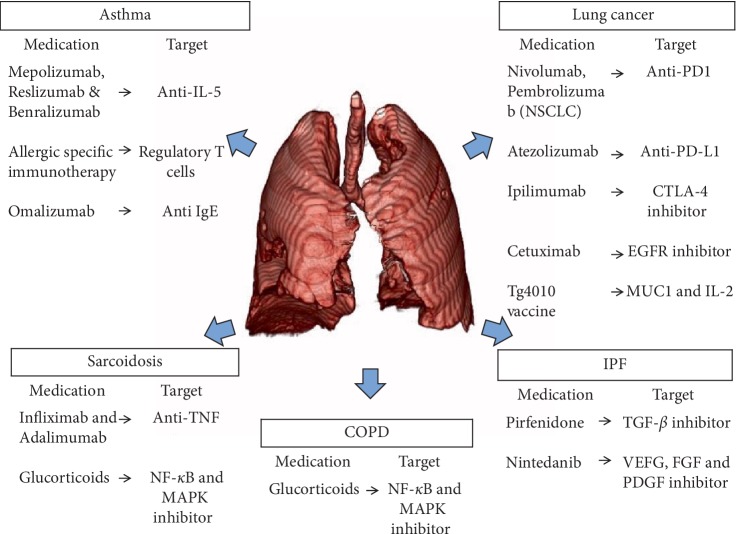
Immunotherapies available to treat different respiratory diseases. VEFG: vascular endothelial growth factor, FGF: fibroblast growth factors, PDGF: Platelet-derived growth factor, MUC1: human glycoprotein mucin 1, CTLA-4: cytotoxic T-lymphocyte-associated protein 4, PD1: programmed cell death protein 1, and PD-L1: programmed death-ligand 1.

Therapeutic efforts have been directed at developing different pharmacologic strategies that target the immune system, particularly regulatory T cells [84, 85]. The modulatory role of regulatory T-cells in allergen-specific immunotherapy has been described in mouse models [85]. Allergen-specific immunotherapy increases the expression of FOXP3 and the production of IL-10 in regulatory cells, while inducing a Th1 response [78, 85, 86]. Immunotherapy also normalizes the relationship between T-regs and other T-cell populations [87]. Other pharmacological strategies have been directed at modulating exaggerated Th2-type response with the use of anti-IL-5 monoclonal antibodies (mepolizumab, reslizumab, and benralizumab) in patients with asthma (Figure 2). Anti-IL-5 monoclonal antibodies have the potential to reduce the incidence of asthma exacerbations by 50% while improving the FEV1 [88]. Other monoclonal antibodies, like anti-immunoglobulin E (Omalizumab) is now recommended in guidelines as an alternative for patients with uncontrolled allergic asthma despite maximal therapy [71]. Lastly, treatment with inhaled corticosteroids in children successfully raises the levels of regulatory T cells in BALF and peripheral blood [82].

## 6. Lung Cancer

Lung Cancer is the first cause of cancer deaths in males and the second in females worldwide [89]. The pathophysiology of this entity is very complex and not entirely understood. However, a growing body of evidence suggests that different branches of the immune system are intimately related. Recent studies have elucidated some of the molecular and biochemical pathways in this entity, highlighting the role of a surrounding microenvironment created by noncancerous cells [90, 91]. Regulatory T cells have a central role in this setting, where they downregulate the antitumor function of cytotoxic CD8+ T cells, promoting tumor growth, survival and anti-apoptotic activity via up-regulation of numerous genes [92]. Abortive activation of immune cells occurs primarily via activation of the nuclear factor kappa-light-chain-enhancer in activated B cells (NF-*κ*B) [93] and through the activity of cyclooxygenase-2 derived products (prostaglandin E2) [94].

The end-product is a tumor-surrounding microenvironment rich in multiple cytokines including TGF-*β*1 and IL-2. Bronchoalveolar lavage fluid samples from patients with primary lung cancer exhibit higher level of TGF-*β*1 as compared to the healthy subjects [95]. Similarly, patients with non-small cell lung cancer (NSCLC) display higher levels of IL-2 as compared to healthy controls [96]. Both IL-2 and TGF-*β*1 are known stimulators of differentiation of Tregs in lung cancer [97]. This stimulus of the Tregs results in subsequent increase in IL-10 and TGF-*β* production, which in turn, inhibit the cytotoxic antitumoral responses. Hence, Tregs are associated with suppression of antitumor immune response, self-tolerance and rapid progression of the disease [98]. Patients with higher proportion of intra-tumoral Tregs are associated with a higher risk of recurrence after tumor resection [90]. A high ratio of stromal FOXP3 to CD3+ cells, favors a pro-tumor environment and predicts cancer progression [99]. Interestingly, patients with low tumor FOXP3 expression and high Treg count have a significantly worse overall survival [100]. These results suggest that tumor FOXP3 expression could be used as a high-fidelity prognostic potential in NSCLC [100].

In direct cell-cell mechanism, Tregs interactions can cause immunosuppression and have been previously described and are considered to play an important role in tumor growth and regulation. Briefly, In the perforin/granzyme apoptosis mechanistic pathway, activated Treg cells attack target cells such as T lymphocytes and APC (antigen presenting cells) through the secretion of perforin and granzyme A. These enzymes are responsible for the formation of pores and hydrolysis of proteins and the ultimate outcome of this interaction is cellular death [101, 102]. Another mechanism of immunosuppression occurs through the interaction between cytotoxic T-lymphocyte-associated antigen 4(CTLA4) and APC. CTLA4+ Treg cells induce the expression of the enzyme 2,3-dioxygenase (IDO) in APC. IDO degrades, and depletes tryptophan levels, causing T-lymphocyte apoptosis [102, 103]. Also, B7-H4 molecule (B7x, B7S1) [104], expressed on lung APC's induced by Tregs is known to cause a stall in the cell cycle of T lymphocytes rendering them inactive [102]. Cytokine mediated responses or cell-cell interaction by the mechanism aforementioned lead to down-modulation of the immune response, which in the context of neoplasia, promotes a microenvironment for unopposed tumor growth and proliferation.

In summary, Tregs suppress the function of various immune cells allowing cancer cell proliferation, through mechanisms that include direct cellular interaction and cytokine-mediated suppression. Increased understanding of Tregs' functional mechanisms will lead to successful clinical trials and the development of Treg-oriented novel therapies.

## 7. Idiopathic Pulmonary Fibrosis

Idiopathic pulmonary fibrosis (IPF) is a progressive lung disease characterized by an increased deposition of extracellular matrix component by fibroblasts (e.g., collagen and fibronectin) that leads to lung parenchymal fibrosis, decreased lung compliance and gas exchange alterations [105, 106]. The pathological processes behind pulmonary fibrosis involve a combination of genetic alterations, intrinsic and extrinsic stressors [107]. Although regulatory T cells are crucial in maintaining immune tolerance and immune homeostasis, but their role in the pathogenesis of IPF remains to be determined [108–111].

Fibrosis is driven mainly by an uncompensated Th2-type immune response, as demonstrated by murine models of lung disease [111]. Treg counts are decreased in the BAL and peripheral blood specimens of IPF patients. Impairment in Treg subpopulations correlates with disease severity, suggesting a critical role for Tregs in the fibrotic process [108, 112]. Common alterations are observed in Treg-derived cytokines including TGF-*β*, TGF-*β*1, and IL-10 [110, 113]. Despite the immunomodulatory role of TGF-*β*, multiples studies have linked this cytokine to an increase in matrix protein synthesis and a decrease in matrix proteinase activity [107, 114–116]. Tregs positive for Sema 7a1 gene induce fibrosis in the TGF-*β*1–exposed murine lung [117]. Similarly, IL-10 down-regulates type I collagen synthesis in human scar tissue-derived fibroblasts, suggesting direct fibrosis inhibition [118, 119]. Experimental mouse models have demonstrated that IL-10 protects against lung fibrosis, even when challenged with bleomycin [120]. However, despite its success in some clinical studies, the mechanism by which IL-10 confers protection from fibrosis remains unclear [110, 121]. Altogether, despite initial evidence of the role of Tregs in IPF, their function in the pro-fibrotic or anti-fibrotic cascades remains to be investigated.

## 8. Conclusion

It is very clear from the experimental and clinical studies that Tregs have a central function in maintaining immune homeostasis in healthy individuals, but they also play a critical role in the physiopathology of some respiratory disease such as sarcoidosis, asthma, IPF, COPD, and lung cancer. The exact mechanism and relationship between Tregs and the aforementioned conditions is still yet to be determined, however, it seems that an imbalance (increase or decrease) in Treg cell count and activity could be key factors responsible for the development of a pathologic state.

Even though, there are dissimilarities between different disease groups, there are some common features of the above-mentioned lung disorders with respect to Tregs. Overall, Tregs plays a critical role in maintaining peripheral tolerance and inhibiting autoimmunity (negative regulation) as well as in stimulating lung tissue repair and well symbiosis (positive regulation). Lastly, few questions that continue to remain open regarding the roles of regulatory T cells in pulmonary illness are: Are the experimental methods used to identify Tregs consistent in all research studies? Are these divergent results perhaps caused by differences in Treg definition or detection? Further research in this area is needed as identification of key molecular pathways would open the door for potential novel therapeutic targets in different respiratory diseases (Figure 2).

## Figures and Tables

**Table 1:** The role of regulatory T cells and different immunological biomarkers in regard to different respiratory diseases. “M” accounts for mouse models and “H” for human models.

Disease: mechanism	sp.	Biomarker studied	Reference
*Asthma*			
T-reg cells from patients with allergic asthma have lower expression of FoxP3, XCL1 and XCR1, leading to suboptimal inhibitory function. Also, impaired chemotactic response to CCL1 correlates with asthma severity.	H	FoxP3	[79, 81, 122]
		XCL1	
		XCR1	
		CCL1	
Increased bronchial density of T-regs, effector T cells, proliferative T cells and activated CD8+ T cells in asthmatics exposed to occupational noxious stimulus shows that both the effector T cells and the inhibitory T-reg, system are activated in asthma.	H	CD4+	[83]
		CD8+	
Airway hyper-reactiveness is modulated by regulatory T cells through induction of TGF-*β*. Regulatory T cells, modulate lung eosinophilia and Th2 response in allergen-induced airway inflammation.	M	TGF-*β*	[77, 123]
		IL-5	
		IL-13	
Drugs targeting IL-5 decrease asthma exacerbation rate by up to 50% in patients with eosinophilic phenotype.	H	IL-5	[124]

*COPD*			
During COPD exacerbations, increased proportions of pro-inflammatory T-reg subpopulations and decreased proportion of suppressive T-regs are observed. Higher Th17 cell counts are also observed, decreasing the T-regs/Th17 ratio. Elevated regulatory T-cells are observed in pulmonary lymphoid follicles.	H	IL-17	[55, 64–66]
		IFN-*γ*	
		TGF-*β*	
		IL-10	
		CD62L	
		FoxP3^+^	
Regulatory T cells are present in lower counts in the small airways of patients with COPD as compared to healthy controls.	H	IL-10	[56]
		IL-17	
		TGF-*β*	
Caveolin-1 plays a crucial role in the imbalance between Th17 and T-regs. Populations in patients with COPD.	H	Cav-1	[62]
		TGF-*β*	
		IL-17	
Peripheral blood T-regs from COPD have an impaired function to suppress CD4+ T-cell activation when stimulated *in vitro*.	H	Foxp3^−^ CD4+	[60]
		CD45RO+	
Proportion of circulating T-regs in COPD patients decreased significantly following long-term treatment with bronchodilators and inhaled steroids.	H	IL-17A	[67]
		IL-8	
		TNF-*α*	
		IL-10	

*IPF*			
Higher expression of Semaphorin 7a is observed on regulatory T cells from individuals with IPF.	H/M	SEMA7A	[117]
		TGF-*β*1	
		IFN-*γ*	
		IL-4	
		IL-10	
		IL-17A	
Individuals with IPF have lower proportions of regulatory T cells in bronchoalveolar lavage and peripheral blood and these have limited inhibitory activity. Low T-reg cell counts are inversely correlated with disease severity.	H	TNF-*α*	[108, 112]
		IFN-*γ*	
		Ki-67	

*Lung cancer*			
Higher proportions of circulating regulatory T-cells are present on lung cancer patients. These are associated to worse clinical outcome.	H	IL-10	[98, 125]

		TGF-*β*	
		IFN-*γ*	
Tumor production of PGE_2_ via COX-2 induces regulatory T cell activity by increasing expression of *FoxP3*. A positive correlation exists between the degree of Treg infiltration and COX-2 expression in NSCLC tumor samples. Recurrence free survival decreases with higher COX-2 expression.	M / H	COX-2 & PGE_2_	[126, 127]
		EP2 & 4 R	
		FoxP3	

*Sarcoidosis*			
Higher counts of Th17 lymphocytes and lower counts of T-reg. Cells are observed in peripheral blood samples of patients with sarcoidosis.	H	ROR-*γ*t	[42]
Circulating regulatory T cell counts are increased in sarcoidosis patients, however these have inefficient suppressive ability. Levels are reduced significantly following corticosteroid therapy.	H	IL-2	[43, 128]
		IL-17A	
		TGF-*β*1	
		IL-6	
		IFN-*γ*	
		IL-10	
		CCL20	
A higher ratio of T-helper to T-suppressor cells are observed in the airways of patients with sarcoidosis. These imbalances lead to granuloma formation. Bronchial T-regs in active sarcoidosis highly express ICOS.	H	ICOS ICOS-L	[38, 39]

## Abbreviations

BAL: Bronchoalveolar lavage
BALF: Bronchoalveolar lavage fluid
CCL1: Chemokine ligand 1
CD: Cluster of differentiation
COPD: Chronic obstructive pulmonary disease
COX-2: Cyclooxygenase 2
CTLA-4: T-lymphocyte antigen 4
COX-2: Cycloxygenase-2
FEV_1_: Forced expiratory volume, on first second
HTERT: Human telomerase reverse transcriptase
IFN-*γ*: Interferon *γ*
IPF: Idiopathic pulmonary fibrosis
FoxP3: Forkhead box P3
IL: Interleukin
MUC5B: Mucin 5B
NF-*κ*B: Nuclear factor kappa-light-chain-enhancer in activated B cells
SCLC: Small cell lung cancer
SFTPC: Surfactant protein C
PB: Peripheral blood
PG E2: Prostaglandin E2
PDGF: Platelet-derived growth factor
Th 1, 2 and 17: T-helper cell type 1, 2 or 17
TGF-*β*: Transforming growth factor *β*
TNF-*α*: Tumor necrosis factor- alfa
XCL1: Chemokine (C motif) ligand 1 (or lymphotactin)
XCR1: XCL1 and XCL2 receptor
PG E_2_: Prostagladin-E2.
